# ID2 promotes survival of glioblastoma cells during metabolic stress by regulating mitochondrial function

**DOI:** 10.1038/cddis.2017.14

**Published:** 2017-02-16

**Authors:** Zhonghua Zhang, Gilbert J Rahme, Pranam D Chatterjee, Matthew C Havrda, Mark A Israel

**Affiliations:** 1 Norris Cotton Cancer Center, Lebanon, NH 03756, USA; 2 Department of Pediatrics, Geisel School of Medicine at Dartmouth, One Medical Center Drive, Lebanon, NH 03756, USA; 3 Department of Medicine, Geisel School of Medicine at Dartmouth, One Medical Center Drive, Lebanon, NH 03756, USA; 4 Department of Molecular and Systems Biology, Geisel School of Medicine at Dartmouth, One Medical Center Drive, Hanover, NH 03755, USA

## Abstract

Tumor cells proliferate in cellular environments characterized by a lack of optimal tissue organization resulting oftentimes in compromised cellular metabolism affecting nutrition, respiration, and energetics. The response of tumor cells to adverse environmental conditions is a key feature affecting their pathogenicity. We found that inhibitor of DNA binding 2 (*ID2*) expression levels significantly correlate with the ability of glioblastoma (GBM)-derived cell lines to survive glucose deprivation. ID2 suppressed mitochondrial oxidative respiration and mitochondrial ATP production by regulating the function of mitochondrial electron transport chain (mETC) complexes, resulting in reduced superoxide and reactive oxygen species (ROS) production from mitochondria. ID2 suppression of ROS production reduced mitochondrial damage and enhanced tumor cell survival during glucose deprivation. Bioinformatics analysis of GBM gene expression data from The Cancer Genome Atlas (TCGA) database revealed that expression of *ID2* mRNA is unique among *ID* gene family members in correlating with the expression of nuclear genes involved in mitochondrial energy metabolism and assembly of mETC. Our data indicate that the expression level of *ID2* in GBM cells can predict the sensitivity of GBM-derived tumor cells to decreased glucose levels. Low levels of ID2 expression in human GBM tissues may identify a clinical group in which metabolic targeting of glycolytic pathways can be expected to have the greatest therapeutic efficacy.

Cancer cells exhibit high rates of glucose uptake and aerobic glycolysis to meet their increased energy, biosynthetic, and redox needs.^1, 2, 3^ Such altered metabolism that enhances tumor cell dependence on glucose for proliferation and survival and is commonly observed in glioblastoma (GBM), the most malignant primary brain tumor, where increased glycolytic rates and poor tumor vasculature lead to low intra-tumoral glucose.^4, 5, 6, 7, 8, 9^

Low glucose has been shown to trigger cell death by promoting mitochondrial reactive oxygen species (ROS) production as a result of impaired mitochondrial homeostasis, a mechanism observed in GBM as well as other tumors.^10, 11, 12^ Suppression of mitochondrial oxygen consumption or reprogramming of metabolism driven by cellular adaptive responses can reduce ROS production and rescue cells from nutrient stress.^5, 13, 14, 15^ Blocking these responses to glucose deprivation is predicted to suppress tumor progression by increasing stress-induced ROS levels and might be efficacious in creating a therapeutic index facilitating therapy.^5, 12, 14, 16^

Members of the inhibitor of DNA binding (*ID*) gene family are key mediators of malignancy that regulate tumor initiation, proliferation, and invasion.^17, 18, 19, 20^ *ID2* is regulated in response to a myriad of stresses including hypoxia, ischemia, AMPK pathway activation, and insulin pathway induction, suggesting a role in adaptive cellular responses to metabolic stress.^21, 22, 23, 24^ Consistent with this hypothesis, *Id2* null mice fail to maintain normal blood glucose levels upon fasting or when fed a low-fat diet and are more sensitive to glucose tolerance testing when compared with wild-type littermates.^25, 26^

We examined the role of ID2 in the cellular response to glucose deprivation, a common metabolic stress in malignant tissues. *ID2* mRNA expression levels in 23 human GBM-derived cell lines correlated with cell survival following glucose deprivation. ID2 suppressed ROS production, enhanced tumor cell survival, and protected mitochondria from damage, during glucose deprivation. Further, we discovered that ID2 contributes to maintenance of mitochondrial membrane potential, oxidative respiration, and mitochondrial electron transport chain (mETC) function. Importantly, a correlation analysis using data from the The Cancer Genome Atlas (TCGA) database indicated that expression of *ID2*, but not other *ID* genes, was associated with expression of genes involved in mitochondrial energy metabolism and mETC assembly. These findings support a pro-survival role for ID2 during metabolic stress that is mediated by its modulation of mitochondrial function and ROS production.

## Results

### ID2 expression in human GBM-derived cell lines correlates with tumor cell survival following glucose deprivation

**Figure 1:**
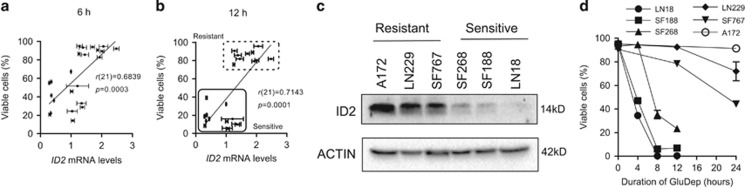
ID2 expression in human GBM-derived cell lines correlates with cellular sensitivity to glucose deprivation (GluDep). (**a**, **b**) Correlation of *ID2* mRNA expression and viability of human GBM-derived cell lines following GluDep for 6 (**a**) and 12 (**b**) hours. The correlation coefficient and significance were determined by the Pearson *r* test. The lines reflect the linear fit of the data shown. (**c**) Expression of ID2 protein in human GBM-derived cell lines detected by immunoblotting. *β*-actin was examined as the loading control. (**d**) Viability of human GBM-derived cell lines following GluDep for the indicated durations

To evaluate whether expression of *ID* genes predicts cellular tolerance to metabolic stress, we measured cell death in 23 human GBM-derived cell lines following glucose deprivation and correlated cellular survival with mRNA expression level of *ID* genes. We found that *ID2* mRNA expression correlated with cellular survival following glucose deprivation (Figures 1a and b). Such correlation was not observed when *ID1* or *ID3 mRNA* was examined (Supplementary Figure S1a–S1d), whereas *ID4* mRNA was weakly correlated (Supplementary Figure S1e, S1f). Thus we focused on the role of ID2 in regulating cellular survival during metabolic stress. We segregated GBM cell lines into two groups (Figure 1b), sensitive and resistant to glucose deprivation, and tested three cell lines from each group for ID2 protein expression that we found to be high in the resistant group and low in the sensitive group (Figure 1c). These cell lines had a differential response to glucose deprivation (Figure 1d).

### ID2 protects human GBM-derived cell lines from cytotoxicity induced by glucose deprivation

**Figure 2:**
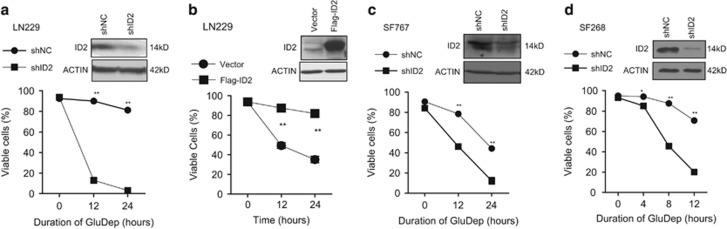
ID2 protects human GBM-derived cell lines from GluDep-induced cytotoxicity. Viability was determined as described in Materials and Methods for LN229(shNC) and LN229(shID2) (**a**), LN229(Vector) and LN229(Flag-ID2) (**b**), SF767(shNC) and SF767(shID2) (**c**), and SF268(shNC) and SF268(shID2) (**d**) following GluDep for the indicated durations. The insets are immunoblots of ID2 protein expression in these cells. *P<0.05; **P<0.01

To study the role of ID2 in regulating cellular survival during metabolic stress, we used shRNA to inhibit ID2 expression in GBM cells that had high ID2 and were resistant to glucose deprivation (Figures 1c and d). LN229 cells with decreased ID2 protein expression (shID2) exhibited reduced cell survival following glucose deprivation compared with control LN229 cells (shNC) (Figure 2a). Increased expression of ID2 protects LN229 cells from death induced by glucose deprivation (Figure 2b). We confirmed this finding in SF767 (Figure 2c) and SF268 cells (Figure 2d), and in an independent experiment by inhibiting the expression of ID2 protein with two different shRNAs targeting human *ID2* mRNA using lentiviruses (Supplementary Figure S2a–S2f). We found that decreased *ID2* expression was associated with reduced tumor cell survival. To verify that this finding was specific for *ID2*, we engineered LN229 cells expressing enhanced levels of *ID1*, *ID2*, *ID3*, or *ID4* and compared their survival during glucose deprivation. Our findings show that LN229 cells engineered to express enhanced levels of *ID2* exhibited increased survival compared with LN229 cells expressing other *ID* genes (Supplementary Figure S3). These data indicate a role for ID2 in tumor cell survival following glucose deprivation.

### ID2 suppresses ROS production induced by glucose deprivation

**Figure 3:**
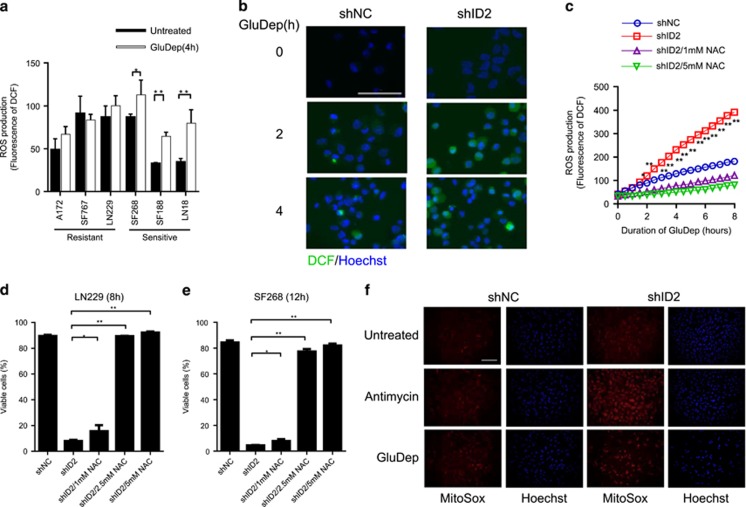
ID2 suppresses GluDep-induced ROS production in human GBM-derived cell lines. (**a**) Intracellular ROS levels in human GBM-derived cell lines following GluDep for 4 h. (**b**) Representative micrographs of DCF fluorescence (green) demonstrating intracellular ROS in LN229(shNC) and LN229(shID2) at the indicated times following GluDep. DNA was counterstained with Hoechst 33258 (blue). Inset scale bar: 200 *μ*m. (**c**) Cumulative ROS production in LN229(shNC) and LN229(shID2) at the indicated times following GluDep and NAC treatment. (**d**) Effect of NAC on the viability of LN229(shNC) and LN229(shID2) following GluDep for 8 h. (**e**) Effect of NAC on the viability of SF268(shNC) and SF268(shID2) following GluDep for 12 h. *P<0.05; **P<0.01; ****P<0.0001. (**f**) Fluorescence (Red) of MitoSOX Red demonstrating mitochondrial superoxide production in LN229(shNC) and LN229(shID2) following GluDep for 4 h or 10 *μ*mol/l antimycin for 30 min

Oxidative stress is a major mediator of cytotoxicity following glucose deprivation.^4, 10, 11^ We used CM-H_2_DCFDA to evaluate ROS production following glucose deprivation in GBM-derived cell lines. Following glucose deprivation, we found that sensitive cells had significantly increased ROS levels, whereas ROS levels did not change in resistant cells (Figure 3a). To determine whether ID2 regulates ROS production following glucose deprivation, we inhibited ID2 expression in LN229 cells and monitored ROS production induced by glucose deprivation (Figure 3b). Prior to glucose deprivation (0 h), the fluorescent signals in both LN229(shNC) and LN229(shID2) cells were undetectable, but following glucose deprivation LN229(shID2) cells invariably had higher fluorescence indicating a higher level of ROS compared with LN229(shNC) cells (Figure 3b). To quantify ROS level changes over time, we monitored ROS accumulation in LN229(shNC) and LN229(shID2) cells following glucose deprivation (Figure 3c). Glucose deprivation induced significantly higher ROS levels in LN229(shID2) (391.0±3.2 RFU, 8 h) than in LN229(shNC) cells (181.3±4.1 RFU, 8 h) (Figure 3c). To test whether suppression of ROS production rescues cell death associated with the loss of ID2 expression following glucose deprivation, we treated cells with *N*-acetyl-L-cysteine (NAC): a free radical scavenger that inactivates ROS.^27^ We found that NAC suppressed ROS accumulation in LN229(shID2) cells (Figure 3c) and significantly enhanced the survival of LN229(shID2) cells following glucose deprivation (Figure 3d).

We extended these observations to SF268 cells, which we treated with either 1 mmol/l or 5 mmol/l NAC in combination with glucose deprivation. We found that NAC significantly enhanced the survival of SF268(shID2) cells (Figure 3e) in a dose-dependent manner following glucose deprivation.

We next determined the source of ROS production by staining cells with MitoSOX Red, which selectively detects mitochondrial superoxide. Antimycin treatment, included here as a positive control, enhances the superoxide production in mitochondria. Glucose deprivation induced brighter fluorescence in LN229(shID2) cells than LN229(shNC), suggesting that ID2 suppresses mitochondrial superoxide production during glucose deprivation (Figure 3f).

### ID2 maintains mitochondrial integrity during glucose withdrawal

**Figure 4:**
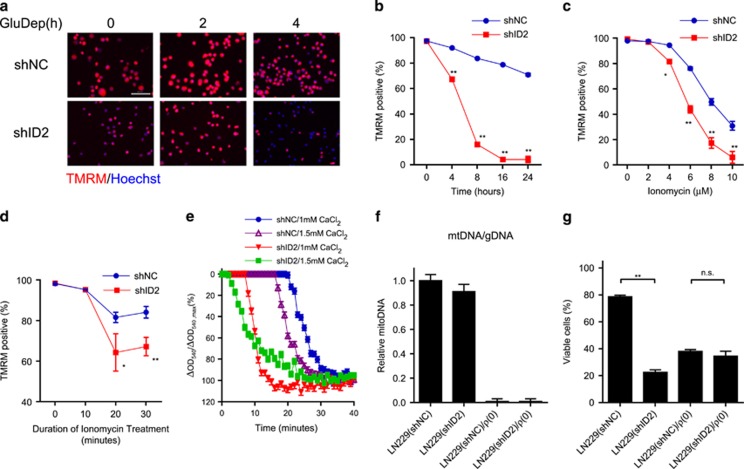
ID2 protects mitochondria from damage following GluDep. (**a**) Representative fluorescent micrographs of mitochondria in LN229(shNC) and LN229(shID2) cells stained by TMRM (red) following GluDep for the times indicated. DNA was counterstained with Hoechst 33258 (blue). Inset scale bar: 200 *μ*m. (**b**) Mitochondrial damage measured by decreased TMRM fluorescence in LN229(shNC) and LN229(shID2) cells following GluDep for the times indicated. (**c**) Mitochondrial damage in LN229(shNC) and LN229(shID2) cells following ionomycin treatment for 30 min at the indicated doses. (**d**) Mitochondrial damage in LN229(shNC) and LN229(shID2) cells following ionomycin treatment (6 *μ*mol/l) for the times indicated. (**e**) *In vitro* calcium-induced swelling of mitochondria isolated from LN229(shNC) and LN229(shID2) cells. The swelling assays were performed three times and representative data are shown as mean±S.D. from four replicates in one assay. (**f**) Relative mitochondrial DNA copy number in LN229(shNC), LN229(shID2), and *ρ*(0) cells. (**g**) Viability of LN229(shNC), LN229(shID2), and *ρ*(0) cells following GluDep for 8 h. *P<0.05; **P<0.01

To assess whether ID2 affects mitochondria integrity during metabolic stress, we evaluated the fluorescent intensity of tetramethylrhodamine methyl ester (TMRM)-stained GBM cells following glucose deprivation. We found that mitochondria required ID2 expression to maintain their integrity following as little as four hours of glucose deprivation (Figures 4a and b). This finding provides evidence that ID2 protects mitochondria from damage induced by metabolic stress.

Another mechanism by which glucose deprivation enhances mitochondrial ROS production and triggers mitochondrial damage is disruption of calcium homeostasis resulting in increased intracellular calcium concentration.^28, 29^ We therefore examined whether ID2 can protect mitochondria from calcium overload induced by glucose deprivation. We treated LN229(shNC) and LN229(shID2) cells with ionomycin, a chemical calcium ionophore,^28^ to increase intracellular free calcium and determined mitochondrial integrity using TMRM staining. We found that ID2 protected mitochondria from damage owing to ionomycin treatment in both a dose- (Figure 4c) and time-dependent manner (Figure 4d).

To further confirm this finding, we isolated mitochondria from LN229(shNC) and LN229(shID2) cells and assessed their sensitivity to exogenous calcium overload in an *in vitro* mitochondrial swelling assay.^30^ We found that mitochondria from LN229(shID2) cells were more sensitive to increased calcium concentration than those from LN229(shNC) cells (Figure 4e). These data provide further evidence that ID2 protects mitochondria from impaired calcium homeostasis induced by glucose deprivation and modulates the response of mitochondria to stress.

Deficiencies in mitochondrial oxidative phosphorylation (OXPHOS) and mETC complexes are known to increase the sensitivity of cancer cells to glucose deprivation.^11, 31, 32^ We examined whether the role of ID2 in regulating mitochondrial function was related to its pro-survival function during metabolic stress. We removed active mitochondria from LN229(shNC) and LN229(shID2) cells using an ethidium bromide-based method to prepare a pooled population of *ρ*(0) cells depleted of mitochondrial DNA, which encodes genes essential for normal mitochondrial function.^11, 33^ These *ρ*(0) cells retained <1% of the mitochondrial DNA present in parental cells (Figure 4f) and exhibited the expected increase in glycolysis, decrease in mitochondrial potential, and dependence on uridine for survival (data not shown).^33^ We examined the viability of *ρ*(0) cells and parental cells following glucose deprivation (Figure 4g). Although ID2 enhanced the survival of LN229 cells with functional mitochondria following glucose deprivation, ID2 lost its protective effect following the removal of mitochondrial DNA from these cells. These suggest that the pro-survival function of ID2 is mediated by its effect on mitochondrial function.

### ID2 maintains mitochondrial membrane potential

**Figure 5:**
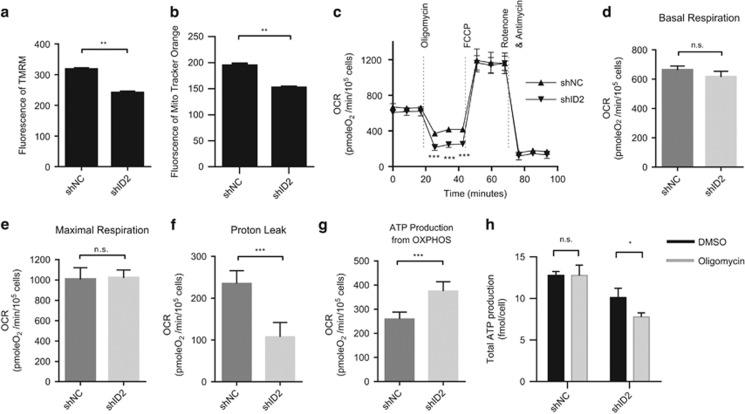
ID2 suppresses mitochondrial oxidative respiration and mitochondrial ATP production. (**a**–**b**) Mitochondrial membrane potential in LN229(shNC) and LN229(shID2) cells was determined by flow cytometric measurement of the mean intensity of TMRM (**a**) or MitoTracker Orange (**b**) fluorescence. (**c**) Mitochondrial oxidative respiration in LN229(shNC) and LN229(shID2) cells determined by the Seahorse XF24 analyzer. The chart of oxygen consumption rates (OCR) versus time as shown is representative of four independent experiments in which five replicates of each assay were evaluated. Basal respiration (**d**), maximal respiration (**e**), proton leak (**f**), and mitochondrial ATP production (**g**) were calculated. (**h**) Mitochondrial ATP production of LN229(shNC) and LN229(shID2) cells in the presence of DMSO or 1 *μ*mol/l oligomycin was determined by ATP luminescence. **P<0.01; ***P<0.001; n.s., no significance

We next explored the possible mechanism by which ID2 exerts a protective effect on mitochondria. We first studied whether ID2 regulates mitochondrial membrane potential, an important indicator of mitochondrial function. We found that decreased ID2 expression significantly reduced the mitochondrial membrane potential in LN229 cells using two mitochondrial potential fluorescent sensors, TMRM and MitoTracker Orange CMTMRos (Figures 5a and b).^34^

We also determined whether ID2 contributes to mitochondrial biogenesis.^35^ We quantified mitochondrial DNA copy number using primers targeted to the mitochondrial NADH dehydrogenase 1 (mt-ND1) gene and normalized to the level of a nuclear gene, lipoprotein lipase. We found that ID2 expression levels had a weak effect on the mitochondrial DNA copy number in LN229 cells (Supplementary Figure S4a). To confirm this finding, we repeated the experiment using primers targeted to the mitochondrial leucine-tRNA gene and the nuclear gene DNA polymerase subunit gamma. No significant difference in mitochondrial DNA copy number was found in these cells (Supplementary Figure S4b). We also examined the effect of ID2 on mitochondrial mass using 10-Nonyl acridine orange (NAO) dye, which selectively binds to mitochondria independent of the mitochondrial membrane potential.^34^ Flow cytometric examination of NAO fluorescence revealed it to be similar in LN229(shNC) and LN229(shID2) cells (Supplementary Figure S4c). These results indicate that ID2 does not affect mitochondrial mass under the conditions of the assay used, and provides strong evidence for ID2 enhancing mitochondrial membrane potential without affecting mitochondrial biogenesis.

### ID2 suppresses mitochondrial ATP production

Mitochondrial membrane potential results from the transport of electrons through the mETC and the pumping of protons across the inner mitochondrial membrane out of the matrix and into the intermembrane mitochondrial spaces. Mitochondrial membrane potential and its proton gradient drives ATP production linking the function of the mETC complexes to mitochondrial OXPHOS.^36^ To examine whether ID2 regulates mitochondrial membrane potential through an effect on mitochondrial OXPHOS, we measured mitochondrial oxidative respiration in LN229 cells using the Seahorse XF24 Extracellular Flux Analyzer and the Cell Mito Stress test kit (Figure 5c). ID2 expression had no obvious effect on either the basal level (Figure 5d) or the maximal capacity (Figure 5e) of oxidative respiration. Decreased ID2 expression did, however, suppress the leakage of protons (Figure 5f) and enhance ATP production in the mitochondria (Figure 5g). These findings indicate that ID2 suppresses mitochondrial OXPHOS resulting in proton retention in the intermembrane space and an enhanced mitochondrial membrane potential that could affect mitochondrial ATP production.

We used an ATP luminescence assay to measure the fraction of ATP production in the presence and absence of oligomycin, an inhibitor of mitochondrial ATP synthase. Oligomycin treatment had no effect on total ATP production in control LN229(shNC) cells, but significantly suppressed ATP production in LN229(shID2) cells (Figure 5h). These data suggest that ID2 can modulate mitochondrial ATP production and switch the source of intracellular energy production.

### ID2 regulates the mETC complexes

**Figure 6:**
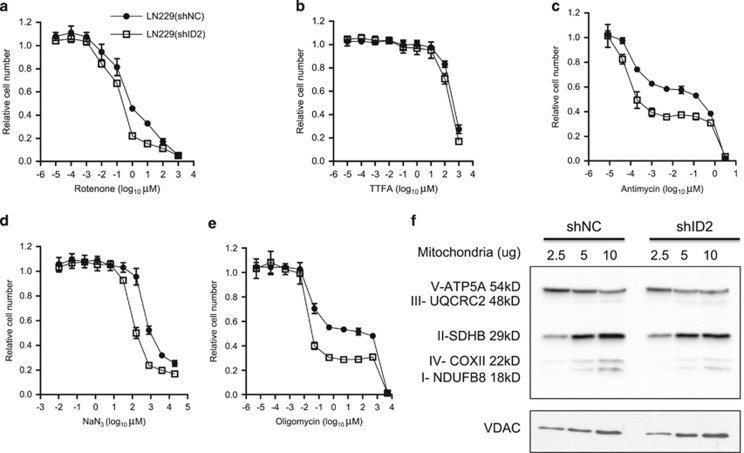
ID2 controls the function of mETC complexes. (**a**–**e**) Dose–response curves of LN229(shNC) (solid circle) and LN229(shID2) (open square) cells to specific inhibitors of individual mETC complexes: blockade of complex I with rotenone (**a**), blockade of complex II with TTFA (**b**), blockade of complex III with Antimycin A (**c**), blockade of complex IV with NaN_3_ (**d**), and blockade of complex V with Oligomycin (**e**). Assays were performed three times and representative data are shown as mean±S.D. from quadruplicates in one experiment. (**f**) Immunobloting of representative subunits of mETC complexes in mitochondria purified from LN229(shNC) and LN229(shID2) cells. VDAC was immunoblotted as the loading reference

Mitochondrial ATP synthesis is driven by the membrane potential and proton gradient that results from the transfer of electrons along the mETC. The mETC includes NADH dehydrogenase (complex I), succinate dehydrogenase (complex II), ubiquinone, bc1 complex (complex III), cytochrome c oxidase (complex IV), and ATPase (complex V).^36^ We sought to determine whether ID2 modulates mitochondrial function and OXPHOS by influencing the activity of mETC complexes. We examined the cytotoxic effect on LN229(shNC) and LN229(shID2) cells of mitochondrial inhibitors: rotenone, TTFA, antimycin, NaN_3_, and oligomycin, which are blockers of the mETC complexes I, II, III, IV, and V, respectively (Figures 6a–e). Comparing the changes in IC_50_, we found that decreased ID2 expression greatly sensitized LN229 cells to complex III (~405-fold) (Figure 6c) and complex V (~730-fold) inhibition (Figure 6e). Decreased ID2 expression also increased the sensitivity of LN229 cells to the blockade of complex I (~4.9-fold) (Figure 6a) and complex IV (~4.8-fold) (Figure 6d). There was no significant change, however, in the sensitivity of these cells to complex II inhibition (Figure 6b).

To determine whether ID2 regulates the assembly of mETC complexes, we examined the expression levels of representative subunit proteins which are liable when their complexes are not well-assembled. ID2 obviously enhanced the expression of subunits assembling complex I and IV, whereas there were no obvious alterations on the integrity of complex II and V(Figure 6f). These findings suggest that ID2 regulates the function of specific mETC complexes thereby modulating mitochondrial OXPHOS and the generation of mitochondrial ROS.

### Expression of ID2, but not other ID genes, correlates with the expression of mitochondrial energy metabolism genes

**Figure 7:**
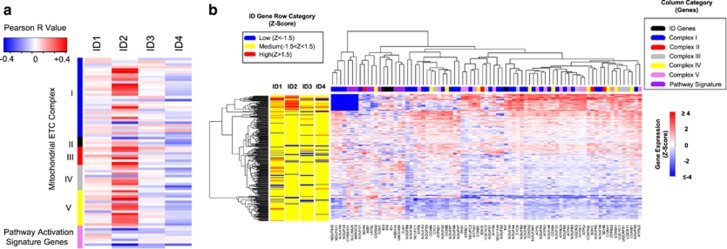
Correlation and cluster analyses of *ID* gene expression and mitochondrial energy metabolism-related gene expression in GBM tumor tissues. (**a**) Heatmap of correlation scores (Pearson *r* value) of mRNA expression levels of *ID* family genes and nuclear genes involved in mitochondrial energy metabolism in GBM tissues that are characterized in TCGA database. (**b**) Hierarchical clustering of mRNA expression (Z-score) of *ID* family genes and mitochondrial energy metabolism-related nuclear genes in GBM tissues charcterized in the TCGA database. Column gene category is color-labeled based on the function and location of proteins encoded by genes we examined. Left panel represents the range of mRNA expression of *ID* family genes in GBM tissues

Our data suggest that ID2 modulates mitochondrial function by affecting the activity of complexes in the mETC. We therefore sought to determine whether ID2 expression can correlate with the expression of genes important for mETC function by analyzing mRNA expression data available in The TCGA database.^37^ We initially performed a Pearson correlation analysis between mRNA expression levels of each member of the *ID* gene family and the expression levels of nuclear genes involved in the assembly and regulation of the mETC complexes. We found that *ID2* expression, but not expression of the *ID1*, *ID3*, or *ID4* genes, correlated positively with the mRNA expression levels of most mETC associated genes (Figure 7a and Supplementary Table S3). Among these, genes encoding proteins of complex I, III, and V, complexes which we found likely to be regulated by ID2 (Figure 6) and which contribute to mitochondrial OXPHOS, mitochondrial membrane potential, and ROS production had the strongest association with *ID2* expression (Figure 7a).^36^ Genes encoding proteins of mETC complex II had a relatively weak correlation with *ID2* expression (Figure 7a), a finding which is consistent with the pattern of the sensitivity of LN229(shNC) and LN229(shID2) cells to treatment with a complex II inhibitor, TTFA (Figure 6b).

In addition, we performed unsupervised clustering of mRNA expression levels of *ID* family genes and mETC genes and found that high levels of *ID2* expression (indicated as the bright red bands in the left column labeled ID2) were associated with high level expression of a subset of the mETC genes (Figure 7b). As expected, among genes that clustered most closely to *ID2* encode were subunits of complex I e.g. *NDUFA5* and *NDUFB2*, complex III, for example, *CYCS*, and complex IV, for example, *NDUFA4* (Figure 7b). ^38^ Further analysis of the cluster associated with high *ID2* revealed that this cluster includes tumors belonging to each of the different molecular subtypes of GBM (Supplementary Figure 5a),^39, 40^ and that loss-of-function alterations in *TP53* were common in this group (39% of patients), which we have previously shown as a mechanism that can drive *ID2* transcription.^41^ There were, however, no overall survival differences between patients represented in this cluster and we identified no clinical parameters that distinguished patients within this cluster(Supplementary Figure 5b). These data provide an independent assessment of GBM tumors that is consistent with our experimental findings of an association between *ID2* expression and mETC structure and function.

## Discussion

Tumor cells are highly anabolic and rely on aerobic glycolysis to meet increased energy and macromolecular substrate requirements for proliferation.^1, 2, 3^ Increased glucose uptake in response to this requirement is the basis FDG-PET scanning and supports the therapeutic potential of targeting tumor metabolism.^42^ One might anticipate that glucose uptake, as measured by FGD-PET positivity would predict the efficacy of such therapy, but we found that neither basal glucose utilization nor tumor aerobic glycolytic rate could predict the sensitivity of GBM-derived cells to glucose depletion (data not shown). This suggests that glycolytic rates may not effectively predict the therapeutic responses to inhibition of glucose metabolism.

Increased glycolysis in tumors, in combination with poor tumor vasculature,^43^ results in lower glucose concentrations in tumor tissue than in adjacent normal tissues.^7, 8^ Although aerobic glycolysis provides sufficient energy and macromolecular substrates for cancer cells to proliferate rapidly when glucose is plentiful, a flexible and adaptive metabolic program allows tumor cells to respond effectively to changes in nutrient availability and stressful metabolic conditions.^5, 12, 14, 15, 16, 31, 32, 42^ Determining how such an adaptive program is regulated may be helpful in predicting the efficacy of metabolic therapy, and could provide insights into the therapeutic resistance of tumor cells to such interventions. Emerging functions for ID2 in regulating energy metabolism may provide insights into pathways that could be targeted for cancer therapy.

Cancer cells are often in environments characterized by hypoxia and nutrient deprivation. Hypoxia inducible factor 1α (HIF1α) regulates tumor metabolism by repressing respiration, while promoting glycolysis. This enables cell survival during stress and promotes tumor cell proliferation.^13, 15, 44^ ID2 is a transcriptional target of HIF1α and enhances the function of HIF1α.^21, 45^ Here, we found that ID2 suppressed mitochondrial ATP production (Figures 5g and h) and regulated mETC (Figures 6a–e). These observations suggest that ID2 can cooperate with other HIF1α target genes such as *PDK1* or *HIGD1A* to redirect glucose from energy production to biosynthesis and regulate cellular responses during hypoxia and nutrient deprivation.^13, 15^

Cancer cells require nuclear-encoded components of mitochondrial OXPHOS to survive during glucose deprivation, whereas they activate glycolytic genes to support cell proliferation when glucose and nutrients are plentiful.^31^ Cellular adaptive response pathways promote the switch of gene expression profiles under nutrient stress.^46^ We found that ID2 enhanced mitochondrial membrane potential (Figures 5a and b) and suppressed mitochondrial OXPHOS and mitochondrial ATP production (Figures 5c, g and h). ID2 protected GBM cells from the blockade of mETC complexes (Figures 6a–e) that link glucose metabolism with energy production from oxidative respiration. To discover the evidence of ID2 activity in GBM tissues, we analyzed the expression of *ID* genes and nuclear-encoded genes critical for the mETC in GBM tissues evaluated in TCGA. *ID2* mRNA levels were associated, uniquely among *ID* family members, with mETC gene expression (Figure 7a). Hierarchical clustering of *ID* and mETC gene expression indicated that *ID2* was closely associated with the expression of a subset of mETC genes (Figure 7b). Genes clustered most closely to *ID2*, such as *NDUFA4*, *NDUFA5*, *NDUFB2*, and *CYCS* encode subunits of complex I, III, and IV, which maintain the proton gradient and mitochondrial membrane potential as well as ATP production.^36^ In combination with impaired glucose utilization observed in *Id2* null mice,^25, 26^ our results suggest that ID2 is a regulator of mitochondrial function that controls cellular adaptive responses during nutrient stress.

Nutrient stress triggers oxidative stress, which originates primarily from mitochondria and is closely related to mitochondrial oxidative respiration and calcium homeostasis.^1, 2, 10, 11, 12, 42, 47^ We found that ID2 inhibits ROS production induced by glucose deprivation (Figures 3b, c and e) thereby reducing mitochondrial damage (Figures 4a and b) and cell death (Figures 3d and f). In addition to decreasing ROS production, we found that ID2 expression suppressed mitochondrial damage and cell death induced by calcium overload (Figures 4c–e), which promotes ROS production and enhances mitochondrial damage.^28, 29^

Glucose depletion results in reverse transport of electrons through the mETC and ROS production, primarily from complex I and III.^7, 36^ We found that ID2 regulates mETC function (Figure 6). Among mETC complexes, ID2 most strongly modulated complexes I, III, IV, and V (Figures 6c–e), which control mitochondrial respiration and energy production. Consistently, ID2 enhanced mETC complex I and IV protein levels (Figure 6f). Bioinformatic analysis of the TCGA database also showed a close correlation between ID2 and components of complex I, III, IV, and V (Figure 7a and b). Our analysis, which utilizes transcriptomic profiling, is limited to its ability to detect patients with high ID2 protein driven by protein stabilization rather than transcriptional upregulation. Albeit our analysis does not address this major ID2 regulatory pathway, we still discovered that ~39% of patients belonging to the high ID2 cluster had loss-of-function mutated TP53, which is consistent with our previous results indicating that mutant TP53 leads to enhanced ID2 expression.^41^ Future analyses that utilize proteomic approaches are more likely to lead to a more thorough identification of patients with high ID2 and could be useful in identifying a larger cohort with metabolic alterations that could show altered survival rates. Collectively, these findings provide evidence of ID2 influencing mitochondrial function and suggest a novel role for the transcription control of mitochondrial related genes, during the adaptive response to nutrient stress.

## Materials and methods

### Cell culture

Human GBM-derived cell lines were maintained in growth medium containing Dulbecco's modified Eagle's medium (DMEM) (Corning, Manassas, VA, USA) with 10% fetal bovine serum (FBS) (HyClone, Logan, UT, USA) and Penicillin/Streptomycin (Corning). SF126, SF188, SF210, SF268, and SF767 cell lines were obtained from the UCSF neurosurgery tissue bank. U87, A172, LN18, and LN229 cells were obtained from the American Type Culture Collection (Manassa, VA, USA). Other cells are from stocks at our laboratory. Glucose-free medium was reconstituted from DMEM (90-113-PB, Corning) supplemented with 10% dialyzed FBS (Life Technologies, Carlsbad, CA, USA), 3.7 g/l of NaHCO_3_, and 4 mmol/l of l-glutamine (Corning). *ρ*(0) cells without mitochondrial DNA were prepared and maintained as previously described.^33^ Retrovirus and lentivirus packaging was performed as previously described.^18, 20^

### Glucose deprivation treatment

Cells were plated into six-well plates at 2 × 10^5^ cells/well and incubated for 16 h in growth medium. Cells were then incubated in glucose-free medium as indicated and evaluated for viability using a propidium iodide (Life Technologies) exclusion assay.

### Suppression and overexpression of ID2 expression in human GBM cells

LN229, SF268, and SF767 cells were transduced with retroviruses derived from pSuperiorRetroPuro vectors (Oligoengine, Seattle, WA, USA) carrying DNA encoding a shRNA that was either a non-targeting negative control (shNC) or a shRNA targeting human *ID2* mRNA (shID2). A172 cells were infected at a multiplicity of infection of 2 with lentiviruses derived from pLKO.1 vectors (Sigma-Aldrich, St. Louis, MO, USA) carrying DNA encoding a non-targeting shRNA control (shNT) or encoding three shRNAs targeting different regions of human *ID2* mRNA (shID2#1, shID2#2). Transduced GBM cells were then selected in puromycin (1 *μ*g/ml, Sigma). shRNA sequences are described in Supplementary Table S1. Overexpression of Flag-tagged human ID2 in GBM cells was performed by transduction with retrovirus derived from pBMN-Flag-ID2 as previously described.^19^ Total RNA isolation, reverse transcription and quantitative PCR (qPCR) were performed as previously described.^18, 20^ Primers used for qPCR are listed in Supplementary Table S2.

### ROS production assay

Cells were incubated in glucose-free medium containing 5 *μ*mol/l of CM-H_2_DCFDA (Life Technologies) and NAC as indicated.^27^ Fluorescence intensity of DCF was continuously monitored every 30 min using a SpectraMax M2e at wavelengths of Ex_485nm_/Em_538nm_ and normalized to signals from untreated cells incubated in parallel in complete growth medium. DCF fluorescence was also measured using flow cytometry (FL1 channel) and fluorescent microscopy (FITC filter). Mitochondrial superoxide production was monitored by incubating cells with MitoSOX Red (Life Technologies) for 30 min after accordingly treatment. Cells were treated with 10 *μ*mol/l of antimycin for 30 min in parallel to induce mitochondrial superoxide production as positive control.

### Mitochondrial DNA, mass, and membrane potential assay

Total genomic DNA was isolated from cultured cells during exponential growth with the DNeasy kit (Qiagen, Valencia, CA, USA). Mitochondrial DNA was detected using qPCR as previously described.^48^ Primer sequences are listed in Table S1. Mitochondrial mass was determined with NAO staining as previously described.^49, 50^

Mitochondrial membrane potential was monitored by flow cytometric analysis of TMRM or MitoTracker Orange CMTMRos (Life Technologies) stained cells.^34, 51^ Mitochondrial membrane potential was measured as the mean fluorescent intensity of TMRM or MitoTracker. Cells with damaged mitochondria were identified as being TMRM negative.

### Isolation of mitochondria, *in vitro* swelling assay, and immunoblot of OXPHOS complexes

Mitochondria from cultured cells were isolated with the Mitochondria Isolation Kit for mammalian cells (ThermoFisher Scientific, Waltham, MA, USA) according to the manufacturer's instructions. Calcium-triggered mitochondrial swelling assays were performed and evaluated as previously described.^30^ Crude mitochondria were further purified with sucrose gradient ultracentrifugation and applied for immunoblotting with antibody cocktails for total human mitochondrial OXPHOS (ab110411, Abcam, Cambridge, MA, USA).

### Mitochondrial oxidative respiration and ATP assay

Cellular oxidative respiration was measured with the Seahorse XF24 Analyzer using the Seahorse Cell Mito Stress Test Kit (Seahorse Bioscience, North Billerica, MA, USA). Assays were performed following treatment with 1 *μ*mol/l of oligomycin, 2 *μ*mol/l of FCCP, and 0.5 *μ*mol/l of rotenone and Antimycin A. The oxygen consumption rate (OCR) was recorded and normalized to cell number. Basal respiration, mitochondrial ATP production, proton leakage, and maximal respiration rates were calculated from OCR data according to the manufacturer's instructions. To detect total ATP, cells were treated with DMSO or 1 *μ*mol/l of oligomycin for 30 min, and intracellular ATP was determined using the ATPlite luminescence assay kit (Perkin Elmer, Waltham, MA, USA).

### Sensitivity to mETC blockade

Cells were plated into 96-well plates at 2000 cells/well in growth medium and incubated for 16 h. Cells were then treated with Rotenone, theonyltrifluoroacetone (TTFA, T27006, Sigma), Antimycin A (A8674, Sigma), NaN_3_ (S8032, Sigma), or Oligomycin (75351, Sigma) at the indicated concentrations for 72 h. Cell numbers were then determined using sulforhodamine B staining.^52^ The IC_50_ was calculated with Prism Graphpad software.

### Bioinformatics analysis

mRNA expression data of all genes in GBM tissues from three different mRNA expression detection platforms (U133 microarray, Agilent microarray, and RNA-seq V2 RSEM) in TCGA database were normalized and standardized (Z-score) to the expression level in diploid tumors (diploid for each gene). These data were downloaded from the cBioPortal for Cancer Genomics, ^53^ and processed to remove the tumors which had not been evaluated for mRNA expression. The final number of patients included was 500. The expression data were then subsetted on R (Version 3.0.3) to include genes involved in mitochondrial energy metabolism and *ID* genes. The selection and classification of mitochondrial energy metabolism genes was based on information from the HUGO Gene Nomenclature Committee (http://www.genenames.org/genefamilies/mitocomplex) website and a gene list from the Human Mitochondrial Energy Metabolism PCR Array (PAHS-008Y, Qiagen). We examined the correlation of mitochondrial gene expression with *ID* gene expression using the Pearson correlation and *R* values (Supplementary Table S3) were plotted as a heatmap (ggplot2 package). For hierarchical clustering, the expression data were transformed to Z-score values between ±4. Genes whose expression levels were undetectable were assigned the value -4 (Supplementary Table S4). These Z-scores were plotted as a heatmap. A dendrogram classification was determined based on complete linkage with Euclidian distance.^37^

### Statistics

The Pearson *r *test was used to evaluate the correlation coefficient between groups. The two-tailed Student's *t*-test was used to evaluate the significance of differences between two groups. Statistical analysis was performed using Prism GraphPad software and R (version 3.0.3). Data are presented as the mean±S.D. from at least three independent experiments when the text does not specifically indicate the origin of the data.
