# Supplementary material for: ID2 promotes survival of glioblastoma cells during metabolic stress by regulating mitochondrial function
Source: Cell Death Dis. 2017 Feb 16;8(2):e2615–. doi: 10.1038/cddis.2017.14 (PMC5386464; doi:10.1038/cddis.2017.14)
Supplement: Supplementary Information [file cddis201714x1.docx]

ID2 Promotes Survival of Glioblastoma Cells during Metabolic Stress by Regulating Mitochondrial Function

Zhonghua Zhang, Gilbert J. Rahme, Pranam D. Chatterjee, Matthew C. Havrda, Mark A. Israel

**Supplementary Figure Legends**

**Figure S1.** Correlation of *ID1*, *ID3*, and *ID4* mRNA expression with the survival of human GBM-derived cell lines following GluDep. mRNA expression was quantified by qRT-PCR of the *ID1* (a, b), *ID3* (c, d), and *ID4* (e, f) genes in human GBM-derived cell lines and plotted against cell viability following GluDep for 6 hours (a, c, e) or 12 hours (b, d, f). Correlation coefficients and significance were determined by Pearson *r* test.

**Figure S2.** Suppression of ID2 with lentiviral shRNAs enhances cell death induced by glucose deprivation. (a to c) Relative *ID2* mRNA expression in glioma cells infected with lentivirus carrying non-target control (shNT) and shRNAs targeting two regions of ID2 mRNA (shID2#1 and shID2#2). (d to f) Cell viability of glioma cells infected with lentiviruses carrying shNT, shID2#1, and shID2#2 after treatments with GluDep for indicated durations.

**Figure S3.** ID2 specifically suppresses tumor cell survival during glucose deprivation in LN229 cells. Cell survival of stable LN229 cell lines infected with viruses engineered to enhance the expression of ID1, ID2, ID3, or ID4.

**Figure S4.** Effects of ID2 expression on mitochondrial DNA copy number and mitochondrial mass in LN229 cells. (a, b) Relative mitochondrial DNA copy number in LN229(shNC) and LN229(shID2) determined by qPCR using primers targeting mt-ND1 (a) or mt-Leu-tRNA (b) and normalized to the level of genomic POLG (a) or LPL (b) gene, respectively. (c) Mitochondrial mass in LN229(shNC) and LN229(shID2) cells was determined by flow cytometric measurement of the mean fluorescence of NAO. Data are presented as mean±SD from three independent experiments. n.s.: no significance.

**Figure S5.** The high ID2 cluster includes patients from different GBM subtypes and does not exhibit survival differences. (a) Pie chart showing the percentage of different GBM molecular subtypes represented in patients belonging to the high ID2 cluster. (b) Kaplan-Meier survival plot showing the overall survival of GBM patients in the high ID2 cluster (in red) compared to the remaining GBM population (in blue).

**Table S1.** Retroviral and lentiviral shRNA vectors.

**Table S2.** Primers for quantitative PCR.

**Table S3.** Correlation score (Pearson *r* test) for mRNA expression of *ID* genes correlated with mitochondrial energy metabolism-related genes in GBM-derived tissues examined in TCGA database.

**Table S4.** Z-score of mRNA expression of *ID* genes and mitochondrial energy metabolism-related genes in GBM-derived tissues examined in TCGA database.

Figure S1

c

b

a

e

d

f

**Figure S1.** Correlation of *ID1*, *ID3*, and *ID4* mRNA expression with the survival of human GBM-derived cell lines following GluDep. mRNA expression was quantified by qRT-PCR of the *ID1* (a, b), *ID3* (c, d), and *ID4* (e, f) genes in human GBM-derived cell lines and plotted against cell viability following GluDep for 6 hours (a, c, e) or 12 hours (b, d, f). Correlation coefficients and significance were determined by Pearson *r* test.

Figure S2.

a

b

c

d

e

f

Figure S2. Suppression of ID2 with lentiviral shRNAs enhances cell death induced by glucose deprivation. (a to c) Relative *ID2* mRNA expression in glioma cells infected with lentivirus carrying non-target control (shNT) and shRNAs targeting two regions of ID2 mRNA (shID2#1 and shID2#2). (d to f) Cell viability of glioma cells infected with lentiviruses carrying shNT, shID2#1, and shID2#2 after treatments with GluDep for indicated durations.

Figure S3

**Figure S3.** ID2 specifically suppresses tumor cell survival during glucose deprivation in LN229 cells. Cell survival of stable LN229 cell lines infected with viruses engineered to enhance the expression of ID1, ID2, ID3, or ID4.

Figure S4

c

b

a

**Figure S4.** Effects of ID2 expression on mitochondrial DNA copy number and mitochondrial mass in LN229 cells. (a, b) Relative mitochondrial DNA copy number in LN229(shNC) and LN229(shID2) determined by qPCR using primers targeting mt-ND1 (a) or mt-Leu-tRNA (b) and normalized to the level of genomic POLG (a) or LPL (b) gene, respectively. (c) Mitochondrial mass in LN229(shNC) and LN229(shID2) cells was determined by flow cytometric measurement of the mean fluorescence of NAO. Data are presented as mean±SD from three independent experiments. n.s.: no significance.

Figure S5

b

a

**Figure S5.** The high ID2 cluster includes patients from different GBM subtypes and does not exhibit survival differences. (a) Pie chart showing the percentage of different GBM molecular subtypes represented in patients belonging to the high ID2 cluster. (b) Kaplan-Meier survival plot showing the overall survival of GBM patients in the high ID2 cluster (in red) compared to the remaining GBM population (in blue).
